# Associations of C-reactive protein and neutrophil-to-lymphocyte ratio with cardiac remodeling, pulmonary hypertension, and survival outcomes in dogs with myxomatous mitral valve disease

**DOI:** 10.14202/vetworld.2026.2279-2292

**Published:** 2026-06-05

**Authors:** Suchada Huttayananont, Chattida Panprom, Sitha Sarikan, Ratiporn Tantisak, Kornnicha Saringkarisate, Soontaree Petchdee

**Affiliations:** 1 Graduate School, Science and Innovation for Animal Health Program, Faculty of Veterinary Medicine, Kasetsart University, Thailand; 2 Kasetsart University Animal Teaching Hospital, Kamphaeng Saen, Faculty of Veterinary Medicine, Kasetsart University, Kamphaeng Saen, Nakhon Pathom, Thailand; 3 Thonglor Pet Hospital, Bangkok, Thailand; 4 Department of Large Animal and Wildlife Clinical Sciences, Faculty of Veterinary Medicine, Kasetsart University, Kamphaeng Saen, Nakhon Pathom, Thailand

**Keywords:** cardiac remodeling, c-reactive protein, dog, myxomatous mitral valve disease, neutrophil-to-lymphocyte ratio, pulmonary hypertension, survival analysis, veterinary cardiology

## Abstract

**Background and Aim:**

Myxomatous mitral valve disease (MMVD) is the most common cardiac disorder in dogs and is frequently associated with progressive cardiac remodeling and pulmonary hypertension (PH). Increasing evidence suggests that systemic inflammation contributes to disease progression; however, the clinical relevance of inflammatory biomarkers in relation to cardiac structural changes and outcomes remains incompletely understood. This study aimed to evaluate the associations between C-reactive protein (CRP), neutrophil-to-lymphocyte ratio (NLR), cardiac remodeling indices, PH, and survival outcomes in dogs with MMVD.

**Materials and Methods:**

A retrospective cohort study was conducted using medical records of 94 client-owned dogs diagnosed with MMVD between 2023 and 2025. Dogs were classified according to the American College of Veterinary Internal Medicine (ACVIM) stages (B1–D). Echocardiographic parameters, including left atrial (LA) volume, LA/Ao ratio, and left ventricular internal diameter, were assessed alongside PH status determined by tricuspid regurgitation velocity. Hematologic parameters and serum CRP concentrations were analyzed. Correlation, regression, receiver operating characteristic (ROC), and survival analyses were performed to determine associations and prognostic value.

**Results:**

LA enlargement was observed in 84.0% of dogs and increased with disease severity. CRP and NLR showed weak but statistically significant positive correlations with indexed LA volume (CRP: r = 0.31, p < 0.01; NLR: r = 0.29, p < 0.05). Neither biomarker demonstrated a significant association with PH. Indexed LA volume, rather than inflammatory biomarkers, was more strongly associated with echocardiographic indicators of PH risk. CRP exhibited moderate diagnostic performance for PH (area under the curve = 0.7517, p < 0.001), whereas NLR showed poor discrimination. Survival analysis revealed that dogs with elevated CRP levels and concurrent PH had significantly reduced survival probabilities. Although NLR increased with disease progression, its prognostic utility remained limited.

**Conclusion:**

Systemic inflammatory biomarkers, particularly CRP, are modestly associated with cardiac remodeling and survival in dogs with MMVD but are not reliable predictors of PH. Echocardiographic indices, especially LA remodeling, remain superior indicators of disease severity and PH risk. While CRP may provide adjunctive prognostic value, its clinical application should be interpreted cautiously alongside established imaging parameters.

## INTRODUCTION

Myxomatous mitral valve disease (MMVD) remains the most frequently encountered cardiac disorder in dogs and typically presents with clinical signs such as coughing and increased respiratory effort. Regardless of the underlying cause, mitral valve disorders generally lead to left atrial (LA) enlargement and, in many cases, pulmonary hypertension (PH) [1, 2]. Echocardiography is considered the gold standard for diagnosing PH in routine veterinary practice [3–6].

In recent years, there has been increasing interest in identifying inflammatory biomarkers for cardiovascular disease, given the well-recognized contributions of inflammation and oxidative stress to the pathophysiology of heart failure [7]. In human medicine, the neutrophil-to-lymphocyte ratio (NLR) has gained attention as a convenient marker of systemic inflammation. Individuals with congestive heart failure (CHF) consistently show elevated NLR values, and several studies have demonstrated that a higher NLR independently predicts all-cause and cardiovascular mortality [8–11]. Veterinary studies have similarly shown that dogs with CHF develop characteristic alterations in their leukocyte profiles, including increased neutrophil and monocyte counts together with reduced lymphocyte levels [12, 13]. In more advanced stages of the disease, increases in acute-phase proteins, particularly C-reactive protein (CRP), and various pro-inflammatory cytokines have also been documented [14, 15]. These findings support the concept that inflammatory processes accompany MMVD progression.

Microscopic examination of diseased mitral valves has revealed infiltration by inflammatory cells and active cytokine signaling within affected tissues [16]. These local changes likely contribute to systemic responses observed in MMVD, including elevations in circulating CRP and shifts in leukocyte ratios such as the NLR. Both CRP and NLR can be obtained from routine hematology and biochemistry panels, making them practical and accessible indicators of generalized inflammatory activity [17, 18]. In human cardiology, similar indices have been associated with a wide spectrum of cardiovascular disorders, ranging from ischemic disease to degenerative valvular conditions [19, 20].

LA volume has also been proposed as a predictor of PH in dogs with MMVD [21], and PH itself remains an important and evolving area of investigation in the context of MMVD progression [22]. However, despite growing evidence linking inflammation with cardiac remodeling, there is still limited integrated information regarding the relationships among CRP, NLR, LA remodeling, ventricular dimensions, and pulmonary vascular changes in dogs with MMVD. In particular, the extent to which inflammatory biomarkers reflect structural cardiac changes and predict PH and clinical outcomes remains unclear in veterinary patients.

The aim of this study was to comprehensively evaluate the clinical relevance of systemic inflammatory biomarkers, specifically CRP and NLR, in dogs diagnosed with MMVD. The study was designed to investigate the associations between these biomarkers and echocardiographic indicators of cardiac remodeling, including LA size, LA/Ao ratio, and left ventricular dimensions, across different stages of MMVD progression.

In addition, the study aimed to determine the relationship between CRP and NLR and the presence as well as severity of PH, as assessed by echocardiographic parameters such as tricuspid regurgitation (TR) velocity and pulmonary artery measurements. Another key objective was to evaluate the diagnostic performance of these inflammatory markers in identifying PH using ROC curve analysis.

Furthermore, the study sought to assess the prognostic significance of CRP and NLR by examining their association with survival outcomes in dogs with MMVD, both independently and in combination with PH status. By integrating hematologic, biochemical, and echocardiographic data, this study aimed to determine whether CRP and NLR can serve as practical and accessible adjunct biomarkers for predicting disease severity, cardiac remodeling, and clinical outcomes in dogs with MMVD.

## MATERIALS AND METHODS

### Ethical approval

This study was conducted in accordance with institutional guidelines for the use of clinical data in research. The investigation used a retrospective, observational design based exclusively on medical records from Kasetsart University Veterinary Teaching Hospital and Thonglor Pet Hospital, and no animals were subjected to additional procedures specifically for research purposes. Therefore, formal ethical approval was not required according to the Institutional Animal Care and Use Committee of Kasetsart University, as the study involved analysis of existing clinical data only.

All dogs included in the study were managed according to standard veterinary clinical practice for MMVD, and all diagnostic procedures, including echocardiography and blood sampling, were performed as part of routine patient care. Written informed consent was obtained from all owners at the time of admission, permitting the use of anonymized clinical data for research and publication purposes.

Animal welfare and confidentiality were strictly maintained throughout the study. All data were handled in a de-identified manner, and no information that could identify individual animals or owners was disclosed. The study adhered to internationally accepted principles for veterinary clinical research and reporting.

### Study design, period, and location

This investigation used a retrospective, observational design based on medical records from the Kasetsart University Veterinary Teaching Hospital and Thonglor Pet Hospital. Medical records dated June 2023 through June 2025 were reviewed consecutively to avoid selection bias and ensure uniform case inclusion during the study period.

### Animals and case selection

Dogs were considered for enrollment if a complete blood count (CBC) was performed within 24 h of an echocardiographic examination confirming MMVD. Inclusion was not restricted by the presence or absence of clinical signs such as coughing, dyspnea, exercise intolerance, or collapse. Animals were selected from those routinely monitored through scheduled physical examinations and echocardiographic follow-ups. Enrollment was not limited by clinical status; dogs with or without cough, respiratory distress, or recent collapse were all considered. Dogs were typically re-evaluated at approximately monthly intervals as part of routine clinical care, however, actual follow-up duration varied among individuals due to differences in disease progression, referral patterns, and the owner. Dogs were managed in accordance with the standard of care, and treatment varied according to disease stage, clinical status, and clinician judgment (e.g., pimobendan, ACE inhibitors, or diuretics). Treatment status was documented.

### Inclusion and exclusion criteria

Medical records were screened for completeness prior to inclusion. Cases missing echocardiographic parameters, CRP values, or CBC variables were excluded using case-wise deletion. No data imputation methods were applied. All analyses were performed using complete datasets only. Dogs were excluded if any non-MMVD cardiac disease, hematologic disorder, infectious or inflammatory condition, or medical treatment known to influence leukocytes, such as corticosteroids, had been recorded within the previous 3 months. To minimize confounding factors related to chronic oral inflammation, each dog underwent a brief oral examination as described previously [23], and dogs with subclinical inflammatory conditions (otitis, dermatitis, dental grade > 2) were excluded.

### Echocardiography protocol

All echocardiographic examinations were performed using a Philips Affiniti 70 ultrasound system (Philips, Bothell, WA, USA) equipped with an S5-1 phased-array transducer (1–5 MHz). Standard right parasternal and apical imaging planes were obtained in accordance with established veterinary echocardiography recommendations [24].

Measured variables included left ventricular internal diameters in diastole (LVIDd) and systole, normalized left ventricular internal diameter in diastole (LVIDdN), left atrial and aortic root dimensions for LA/Ao ratio, LA volume assessed using the Simpson biplane method, transmitral inflow velocities (E/A ratio), tissue Doppler velocities at the lateral mitral annulus, and main pulmonary artery diameter. When feasible, measurements were averaged over three consecutive cardiac cycles. LVIDdN was calculated using standard allometric scaling:

LVIDdN = LVIDd/Body weight (BW)^0.294^

where LVIDd is measured in centimeters and BW in kilograms.

**Figure 1:**
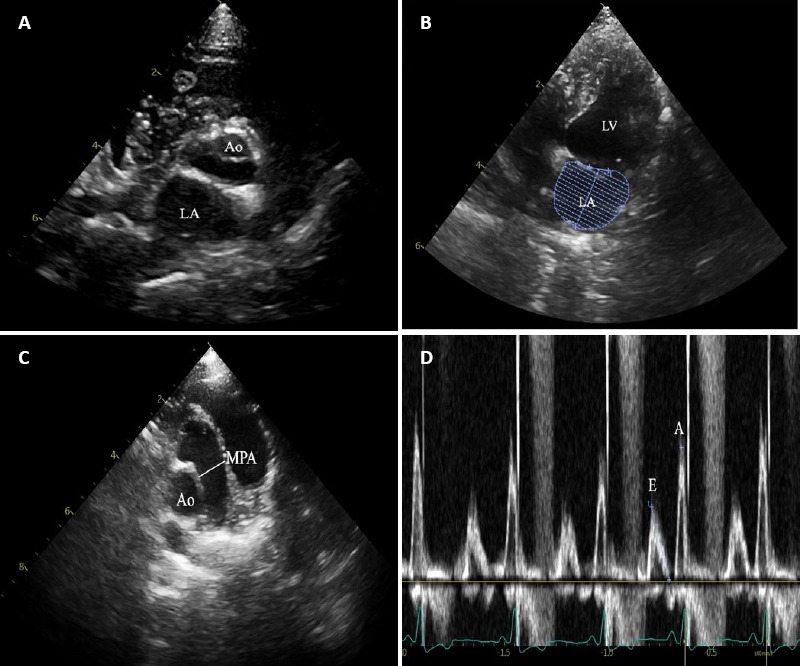
Echocardiographic assessment in dogs with myxomatous mitral valve disease (MMVD). Representative two-dimensional images showing (A) short-axis view, (B) left apical two-chamber view, (C) apical cranial view at the aortic and pulmonic valve level, and (D) pulsed-wave Doppler echocardiography of transmitral flow. Ao = Aorta, LA = Left atrium, LV = Left ventricle, MPA = Main pulmonary artery.

Diagnosis of MMVD was based on characteristic two-dimensional and Doppler findings, including mitral valve leaflet thickening, redundancy, prolapse, and associated regurgitant flow. Disease staging followed the ACVIM 2019 Consensus Guidelines [25]. Left atrial enlargement was assessed using the LA/Ao ratio and indexed LA volume (Figure 1).

PH was evaluated according to the ACVIM 2020 Consensus Statement [26]. PH was defined by a TR velocity > 3.0 m/s (estimated systolic pressure gradient ≥ 36 mmHg). Doppler interrogation was standardized by optimizing transducer position to minimize the angle, with an alignment error maintained below approximately 20° whenever technically feasible. Right-sided chamber enlargement and pulmonary artery dilation were recorded as supportive findings but were not used as primary diagnostic criteria. All echocardiographic studies were performed by experienced clinicians; inter-observer variability was not assessed.

### Hematologic and biomarker assessment

Blood samples were obtained from the cephalic vein and collected into EDTA tubes (BD Vacutainer, NJ, USA) for CBC analysis and into serum separator tubes (BD Vacutainer) for biochemical testing. Sampling was performed during morning hospital hours to minimize diurnal variation. When clinically appropriate, dogs were fasted for at least 6–8 h prior to blood collection.

CBC analysis was performed within 1 h of sampling using an automated hematology analyzer (IDEXX ProCyte Dx, IDEXX Laboratories, Westbrook, ME, USA). The analyzer was calibrated daily according to the manufacturer’s quality-control protocol. Parameters recorded included total white blood cell count, absolute and relative neutrophil and lymphocyte counts, monocyte count, and platelet count. The NLR was calculated as the absolute neutrophil count divided by the absolute lymphocyte count.

Serum CRP concentrations were measured using an immunoturbidimetric assay (IDEXX Catalyst CRP Test, IDEXX Laboratories, Westbrook, ME, USA) at a single accredited laboratory to avoid inter-laboratory variability. The assay is validated for canine samples. According to the manufacturer, the analytical measurement range is 5–200 mg/L, with a lower detection limit of 5 mg/L. The intra-assay and inter-assay coefficients of variation are <5% and <10%, respectively. The reference interval for clinically healthy dogs provided by the manufacturer is <10 mg/L. All analyses were performed in accordance with the manufacturer’s instructions.

### Statistical analysis

Statistical analyses were performed using GraphPad Prism version 10 (GraphPad Software, San Diego, CA, USA). Data distribution was evaluated using the Shapiro–Wilk test. Normally distributed variables are presented as mean ± standard deviation (SD), whereas non-normally distributed variables are reported as median.

Comparisons between two groups were conducted using the unpaired Student’s t-test for normally distributed data or the Mann–Whitney U test for non-parametric data. Comparisons among MMVD stages were performed using one-way analysis of variance (ANOVA) followed by Tukey’s post hoc test when parametric assumptions were satisfied.

Associations between inflammatory biomarkers (CRP and NLR) and echocardiographic parameters were assessed using Pearson or Spearman correlation coefficients, as appropriate based on data distribution. Correlation coefficients (r) with corresponding 95% confidence intervals (CI) were reported. Variables showing significant associations in univariate analysis were further evaluated using linear regression models.

Diagnostic performance for detecting PH was assessed using ROC curve analysis. The area under the curve (AUC) with 95% CI was calculated. Optimal cutoff values for CRP and NLR were determined using the Youden index (Youden index = sensitivity + specificity − 1).

CRP and NLR were analyzed as continuous variables in correlation, regression, and ROC analyses. For survival analysis, biomarkers were dichotomized according to the ROC-derived Youden index cutoffs to generate Kaplan–Meier survival curves. Survival distributions were compared using the log-rank test. A two-tailed p < 0.05 was considered statistically significant.

Survival analysis included only dogs with available follow-up data and complete biomarker measurements. Dogs lost to follow-up or deceased from non-cardiac causes were excluded at the time of last contact. A p < 0.05 was considered statistically significant.

## RESULTS

### Population characteristics and MMVD staging

Ninety-four client-owned dogs with a diagnosis of MMVD were included. The median age was 11 years. Based on ACVIM staging, 8 dogs were classified as stage B1, 43 as stage B2, 38 as stage C, and 5 as stage D. Small-breed dogs comprised most of the study population. Pomeranians (n = 32) and Chihuahuas (n = 27) were the most frequently represented breeds, followed by Shih Tzus (n = 13). The remaining dogs included Poodles (n = 6), Yorkshire Terriers (n = 3), mixed breeds (n = 7), and several other breeds represented by one or two individuals. Because of the small number of dogs in some breed categories, breed-based comparisons were not performed. Dogs were divided according to PH status using TR velocity. Dogs with TR velocity ≤ 3.0 m/s were classified as non-PH, and those with TR velocity >3.0 m/s were classified as PH. Cardiac remodeling was evident from stage B2 onward. Both LA/Ao ratio and LVIDdN were higher in stage B2 compared with stage B1, with further increases observed in stages C and D. General characteristics, hematologic findings, and echocardiographic measurements are summarized in Tables 1–4.

**Table 1:** Clinical characteristics of the study animals.

Parameters	Total (N = 94)	Stage B1 (N = 8)	Stage B2 (N = 43)	Stage C (N = 38)	Stage D (N = 5)	p-value
Age (years)	11.18 ± 2.90	9.00 ± 3.00	10.74 ± 2.81	12.01 ± 2.80	12.00 ± 1.10	0.0662
Body weight (kg)	5.04 ± 3.00	7.39 ± 3.84	4.95 ± 2.70	4.28 ± 1.70	7.70 ± 6.24	0.0253
Male (number, %)	44/94 (46.80%)	3/8 (37.5%)	22/43 (51.16%)	18/38 (47.37%)	1/5 (20.0%)	–

MMVD = Myxomatous mitral valve disease, Data are presented as mean ± SD for continuous variables and number (%) for categorical variables, p-values were calculated using one-way analysis of variance across ACVIM stages (B1–D), Tukey’s post hoc test identified a significant difference in body weight between stage B1 and stage C (p < 0.05).

**Table 2:** Hematologic and biochemical profiles of dogs with different stages of myxomatous mitral valve disease.

Parameters	Total (N = 94)	Stage B1 (N = 8)	Stage B2 (N = 43)	Stage C (N = 38)	Stage D (N = 5)	Reference value
WBC (10³/µL)	13.09 ± 5.86	12.08 ± 5.41	12.84 ± 5.83	13.72 ± 6.27	11.77 ± 1.92	5.0–14.1
Neutrophils (%)	71.34 ± 17.80	84.55 ± 4.45	72.67 ± 15.21	68.06 ± 21.92	71.20 ± 7.24	60–77
Lymphocytes (%)	16.75 ± 6.64	8.25 ± 0.25	16.81 ± 6.88	17.45 ± 6.35	17.70 ± 4.10	12–30
Absolute neutrophils (10³/µL)	9.34	10.21	9.33	9.34	8.38	2.5–8.5
Absolute lymphocyte (10³/µL)	2.19	1.00	2.16	2.39	2.08	0.4–2.9
NLR (N = 65)	5.44 ± 3.84	5.32 ± 3.19	5.48 ± 3.82	5.46 ± 4.23	4.39 ± 1.60	3.0–4.0
CRP (mg/L) (N = 66)	16.41 ± 30.33	4.42 ± 2.98	13.43 ± 24.49	17.04 ± 34.63	39.76 ± 5.28	<10
BUN (mg%)	35.99 ± 24.83	32.17 ± 14.87	31.31 ± 23.98	40.98 ± 26.57	39.75 ± 23.16	10.0–30.0
Creatinine (mg%)	1.41 ± 1.77	1.27 ± 0.89	1.00 ± 0.53	1.60 ± 1.54	4.23 ± 5.65	0.5–1.5
ALT (U/L)	87.47 ± 9.79	163.40 ± 45.42	84.11 ± 16.28	77.10 ± 9.46	88.75 ± 16.63	17–95
ALP (U/L)	116.38 ± 9.41	152.75 ± 33.71	122.79 ± 14.96	106.29 ± 11.47	95.67 ± 17.56	20–150

Absolute neutrophil and lymphocyte counts were retrospectively calculated using total WBC and differential percentages, values represent approximations, WBC = White blood cell, NLR = Neutrophil-to-lymphocyte ratio, CRP = C-reactive protein, BUN = Blood urea nitrogen, ALT = Alanine transaminase, ALP = Alkaline phosphatase.

**Table 3:** Echocardiographic parameters of dogs with different stages of myxomatous mitral valve disease.

Parameters	Stage B1 (N = 8)	Stage B2 (N = 43)	Stage C (N = 38)	Stage D (N = 5)	p-value
LA/Ao ratio	1.58 ± 0.22ᵃ	1.78 ± 0.23ᵃ	1.98 ± 0.40ᵇ	2.74 ± 0.92ᶜ	<0.0001
LA volume (mL)	5.39 ± 3.83ᵃ	5.13 ± 3.01ᵃ	6.97 ± 5.33ᵃᵇ	10.47 ± 8.90ᵇ	0.1058
MPA (cm)	0.87 ± 0.30ᵃ	1.12 ± 0.23ᵇ	1.11 ± 0.28ᵇ	1.58 ± 0.52ᶜ	0.0004
MPA/Ao	0.93 ± 0.09	1.08 ± 0.18	1.15 ± 0.19	1.34 ± 0.19	0.0842
MR volume (mL)	0.90 ± 0.60	0.98 ± 1.97	2.04 ± 2.69	4.10 ± 5.52	0.0564
IVSd (cm)	0.65 ± 0.12	0.58 ± 0.13	0.55 ± 0.16	0.70 ± 0.30	0.1172
LVPWd (cm)	0.59 ± 0.12	0.57 ± 0.13	0.54 ± 0.15	0.64 ± 0.17	0.3881
LVIDd (cm)	2.32 ± 0.58ᵃ	2.26 ± 0.65ᵃ	2.64 ± 0.78ᵇ	3.76 ± 1.62ᶜ	0.0008
LVIDdN (cm)	1.48 ± 0.11	1.50 ± 0.30	1.80 ± 0.41	2.12 ± 0.49	0.5730
FS (%)	39.18 ± 7.19	31.34 ± 6.55	34.01 ± 10.05	34.10 ± 6.53	0.1030
MV E/A ratio	1.17 ± 0.47ᵃ	0.77 ± 0.21ᵇ	1.01 ± 0.47ᵃᵇ	1.33 ± 0.69ᵃ	0.0017
IVRT (ms)	50.00 ± 0.01	60.00 ± 0.02	70.00 ± 0.01	80.00 ± 0.00	0.0565

LA = Left atrium, Ao = Aorta, MPA = Main pulmonary artery, MR = Mitral regurgitation, IVSd = Interventricular septum in diastole, LVPWd = Left ventricular posterior wall in diastole, LVIDd = Left ventricular internal diameter in diastole, LVIDdN = Normalized LVIDd, FS = Fractional shortening, MV E/A = Mitral inflow ratio, IVRT = Isovolumic relaxation time.

**Table 4:** Comparison of dogs with pulmonary hypertension (PH) and non-PH groups.

Variables	PH (N = 49)	Non-PH (N = 45)	p-value
Age (years)	11.82 ± 2.69ᵃ	10.48 ± 2.96ᵃ	0.0237
Body weight (kg)	4.75 ± 2.78	5.35 ± 3.20	0.3334
Sex (male, %)	42.85	51.11	–
Stage B1 (%)	2.04	15.55	–
Stage B2 (%)	36.74	57.78	–
Stage C (%)	51.02	26.67	–
Stage D (%)	10.20	0	–
LA/Ao ratio	1.98 ± 0.49ᵃ	1.80 ± 0.35ᵃ	0.0449
LVIDdN (cm)	1.74 ± 0.46ᵃ	1.56 ± 0.28ᵃ	0.0257
FS (%)	31.07 ± 7.29ᵇ	35.51 ± 9.04ᵇ	0.0100
TR velocity (m/s)	4.80 ± 3.86ᶜ	2.44 ± 0.30ᶜ	0.0001
Pimobendan (%)	97.96	84.44	–
ACE inhibitor (%)	4.08	26.67	–
Furosemide (%)	61.22	26.67	–
Spironolactone (%)	97.96	84.44	–

LA = Left atrium, Ao = Aorta, LVIDdN = Normalized left ventricular internal diameter in diastole, FS = Fractional shortening, TR = Tricuspid regurgitation. Data are presented as mean ± SD, superscripts indicate significant differences between groups.

### Study limitations related to dataset

This study did not include a healthy control group. Biomarker comparisons were therefore limited to differences among MMVD stages and between PH and non-PH groups. Information regarding body condition score and subclinical inflammatory conditions was not consistently available in the medical records and was not included in the analysis.

### Hematologic findings

Total leukocyte counts remained within the reference interval across all MMVD stages (Table 2). Neutrophil percentages were highest in stage B1 (84.55 ± 4.45%) and exceeded the upper reference range in this group, whereas stages B2, C, and D showed mean values within or near the reference interval. Lymphocyte percentages were lowest in stage B1 (8.25 ± 0.25%), which is below the reference range. However, other stages demonstrated mean values within reference limits.

### Echocardiographic findings

**Figure 2:**
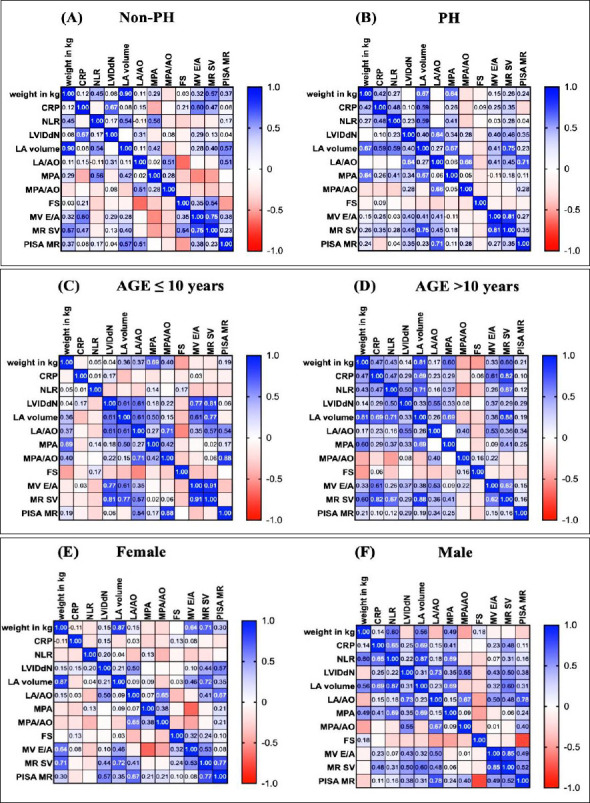
Correlation matrix of inflammatory biomarkers and echocardiographic variables among dogs with mitral valve disease. PH = Pulmonary hypertension, non-PH = Non-pulmonary hypertension, LA = Left atrium, Ao = Aorta, IVSd = Interventricular septum in diastole, LVPWd = Left ventricular posterior wall in diastole, LVIDd = Left ventricular internal diameter in diastole, IVSs = Interventricular septum in systole, LVPWs = Left ventricular posterior wall in systole, LVIDs = Left ventricular internal diameter in systole, FS = Fractional shortening, PV Vmax = Pulmonary valve maximum blood velocity, AV Vmax = Aortic valve maximum blood velocity.

Echocardiographic measurements for all dogs are presented in Table 3. Several structural variables differed among MMVD stages. The LA/Ao ratio increased progressively with disease severity and differed significantly among groups (p < 0.0001). Dogs in stages C and D had higher LA/Ao values compared with stages B1 and B2. Absolute LA volume tended to be greater in advanced stages; however, the overall difference was not statistically significant (p = 0.1058). Left ventricular enlargement was reflected by a significant increase in LVIDd across stages (p = 0.0008), with the largest values observed in stage D. LVIDdN did not differ significantly among groups (p = 0.5730). Pulmonary artery measurements showed stage-related changes. MPA diameter increased significantly with advancing stage (p = 0.0004). Although the MPA/Ao ratio showed a gradual increase from stage B1 to stage D, this did not reach statistical significance (p = 0.0842). Mitral inflow E/A ratio differed significantly among stages (p = 0.0017). No significant differences were identified for IVSd, LVPWd, fractional shortening, MR volume, or Isovolumic Relaxation Time (IVRT). Correlation analyses between CRP and echocardiographic parameters are shown in Figures 2A–F.

### CRP and echocardiographic correlations stratified by PH, age, and sex

Table 4 compares dogs with and without PH, which was defined as TR velocity > 3.0 m/s. Statistical comparisons between groups are also provided within the table. When dogs were categorized according to PH status (TR velocity > 3.0 m/s vs. ≤ 3.0 m/s), correlations between CRP and selected cardiac variables were stronger in the PH group. In dogs with PH, CRP was moderately correlated with LA volume (r = 0.59) and MPA diameter (r = 0.59). In contrast, these associations were weak in dogs without PH (r = 0.08 and r = 0.17, respectively). Age-stratified analysis demonstrated minimal correlations between CRP and echocardiographic measurements in dogs ≤ 10 years of age (correlation coefficients generally < 0.20). In dogs > 10 years, CRP showed moderate positive correlations with LA volume (r = 0.37), LVIDdN (r = 0.57), and MV E/A ratio (r = 0.38). Sex-based analysis revealed weak correlations in females. In males, CRP was moderately correlated with LA volume (r = 0.57), LVIDdN (r = 0.56), and MV E/A ratio (r = 0.39).

### CRP and NLR in relation to PH and MMVD stage

**Figure 3:**
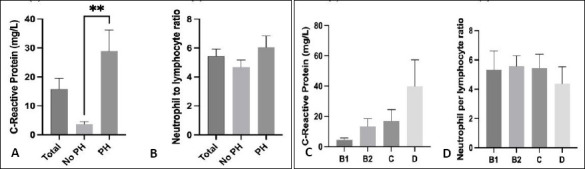
Serum concentrations of (A) C-reactive protein (CRP) and (B) neutrophil-to-lymphocyte ratio (NLR) in dogs with and without pulmonary hypertension (PH), and (C) CRP and (D) NLR across MMVD stages B1, B2, C, and D. **p < 0.01.

Dogs with PH had significantly higher serum CRP levels than those without PH did (p < 0.01). Mean CRP levels in the PH group were greater than 30 mg/L and remained below 10 mg/L in the non-PH group (Figure 3A). These results indicate a strong association between elevated CRP levels and the presence of PH. Although the NLR was greater in the PH group than in the non-PH group, the difference was not statistically significant. The average NLR (Figure 3B) indicated that the NLR may not be a sensitive marker for distinguishing PH in this study population. The CRP levels gradually increased across the advancing stages of heart disease (B1 < B2 < C < D), and the highest values were observed in stage D (Figure 3C). However, no statistically significant difference in CRP levels among the groups was observed. The NLR values were relatively consistent across all stages (B1 to D), with no significant differences observed (Figure 3D). These findings suggest that systemic inflammation may not change significantly with increasing disease stage.

### Diagnostic performance of CRP and NLR

**Figure 4:**
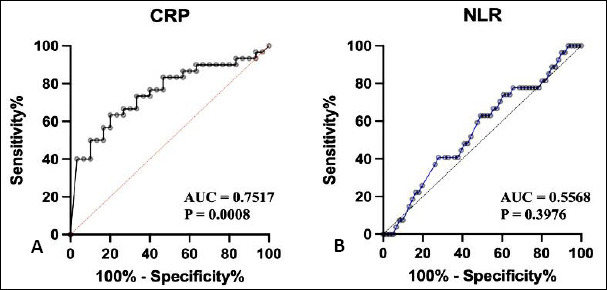
Receiver operating characteristic curves evaluating C-reactive protein (CRP) and neutrophil-to-lymphocyte ratio (NLR) for discrimination of pulmonary hypertension in dogs with myxomatous mitral valve disease. (A) CRP demonstrated moderate diagnostic accuracy with an area under the curve (AUC) of 0.7517 (p = 0.0008). (B) NLR showed limited discriminatory ability with an AUC of 0.5568 (p = 0.3976).

CRP showed an AUC of 0.7517 (p = 0.0008), as shown in Figure 4A, indicating that CRP has statistically significant discriminatory power. However, NLR (Figure 4B) exhibited poor diagnostic performance, with an AUC of 0.5568 and a non-significant p = 0.3976.

### Survival analysis

**Figure 5:**
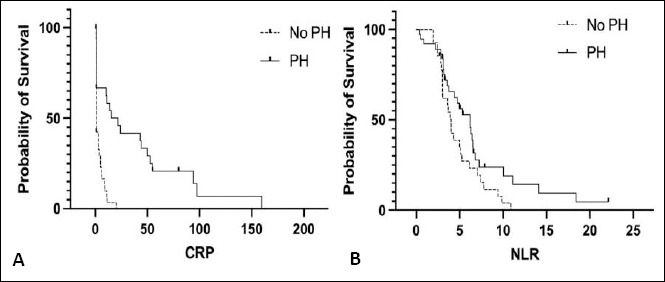
Kaplan–Meier survival curves illustrating survival probability according to (A) C-reactive protein (CRP) and (B) neutrophil-to-lymphocyte ratio (NLR) in dogs with myxomatous mitral valve disease (MMVD), stratified by pulmonary hypertension (PH) status defined by tricuspid regurgitation (TR) velocity > 3.0 m/s and non-PH status defined by TR velocity ≤ 3.0 m/s. In both analyses, dogs with PH consistently had lower survival probabilities than non-PH dogs across increasing CRP and NLR values.

Dogs with PH (solid line) had a lower survival probability with increasing CRP levels than dogs in the group without PH (dashed line), as shown in Figure 5. Survival analysis was performed according to PH status and stratified by CRP and NLR concentrations. When stratified by CRP (Figure 5A), dogs with PH showed shorter survival times compared with dogs without PH at comparable CRP concentrations. Increasing CRP levels were associated with a progressive decline in survival probability in both groups, with a steeper reduction observed in dogs with PH.

Similarly, when stratified by NLR (Figure 5B), survival probability decreased as NLR increased. Dogs with PH consistently demonstrated lower survival probabilities across NLR values compared with dogs without PH. These findings indicate that higher CRP and NLR values are associated with reduced survival, particularly in dogs with concurrent PH.

## DISCUSSION

### Inflammatory biomarkers and MMVD progression

This study explored common hematologic and inflammatory indicators in dogs with different stages of MMVD and demonstrated clear relationships between biomarker changes and disease severity. Although MMVD is traditionally viewed as a degenerative valve disorder causing mechanical and hemodynamic alterations, the results here suggest that systemic inflammation plays a meaningful, and perhaps underappreciated, role in disease progression. One notable trend was the steady rise in NLR as the disease advanced. In the present study, dogs with more advanced MMVD demonstrated higher inflammatory biomarker levels and altered leukocyte profiles. This increase likely reflects chronic activation of sympathetic and neurohormonal pathways together with persistent low-grade inflammation, both of which have been consistently reported in chronic heart disease and CHF across species [27]. Lymphocyte reductions observed in later stages may result from stress-related redistribution, while the rise in neutrophils may be driven by inflammatory processes associated with pulmonary congestion or tissue hypoxia.

CRP concentrations also tended to rise as the disease worsened, reaching their highest values in dogs with clinical signs of heart failure. This pattern is consistent with previous work showing that CRP is influenced by myocardial stretch, neurohormonal activity, and secondary inflammatory pathways [28]. However, NLR showed a tighter association with echocardiographic indices, particularly LA enlargement, than CRP. One explanation may be that NLR changes more rapidly in response to acute hemodynamic stress, whereas CRP may reflect a broader or slower inflammatory response.

### Relationship between NLR, CRP, and LA remodeling

Additionally, NLR demonstrated a positive association with both CRP levels and LA volume, supporting its usefulness as a practical marker for estimating the severity of MMVD. LA enlargement is a sensitive and early marker of chronic volume overload in MMVD. Because LA remodeling precedes left ventricular dilation in many dogs, using LA size as the primary determinant for stage B2 classification provides a practical, pathophysiologically relevant approach, particularly in retrospective clinical datasets.

When echocardiographic variables were compared across MMVD stages, LA volume showed a progressive increase from stage B to stages C and D, consistent with disease severity. However, it is important to interpret these findings cautiously. Although post hoc pairwise comparisons suggested greater LA volume in stage C and D dogs compared with stage B dogs, the overall ANOVA did not reach statistical significance. In the absence of a significant global test, statistically significant post hoc p-values may reflect inflated Type I error rather than true biological differences. Therefore, these findings should be considered exploratory and interpreted in conjunction with effect sizes and clinical relevance rather than isolated p-values.

Cardiac remodeling was also reflected by progressive increases in LA/Ao ratio and LVIDdN, with values exceeding reference thresholds in later stages [29]. In addition, the average LVIDdN of the B2 group in our population was slightly below the theoretical threshold (1.50 ± 0.30). The LVIDdN observed in this cohort was slightly lower than the commonly applied cutoff value of 1.7 used for ACVIM stage B2 classification. This finding may reflect population heterogeneity, disease stage distribution, or treatment effects in dogs undergoing routine clinical follow-up, and highlights the importance of interpreting structural indices within the broader clinical and echocardiographic context.

### Pathophysiological role of inflammation in MMVD

Heart failure has recently been recognized as not only a hemodynamic disorder but also a disorder characterized by inflammatory and immune dysregulation. Inflammation contributes to myocardial injury and complications such as endothelial dysfunction and cardiac cachexia [30]. Neutrophils release inflammatory mediators that may damage the vascular endothelium, whereas lymphopenia, potentially caused by elevated corticosteroids or neurohormonal shifts in heart failure, is associated with poor outcomes. In dogs, reductions in total lymphocyte counts and lymphocyte subsets have been observed more frequently in canine patients with severe CHF. Platelets also participate in the inflammatory cascade and thrombus formation, particularly in conditions such as mitral valve disease, where altered flow dynamics can influence platelet behavior.

Numerous veterinary studies have evaluated CRP in inflammatory and neoplastic conditions, and only recently has attention turned toward its relevance in canine cardiac disease. In this study, increased neutrophil counts and decreased lymphocyte levels (within the normal range) were observed in dogs with MMVD and concurrent PH, contributing to elevated CRP levels and NLR values [31, 32]. The higher average CRP and NLR values observed here may be attributed to the smaller sample size compared with previous reports. Notably, CRP levels were significantly elevated in dogs with PH, in agreement with findings from human heart failure studies, where CRP is considered a prognostic factor.

### Leukocyte dynamics across MMVD stages

Hematologic comparisons across disease stages revealed stage-dependent alterations in leukocyte profiles. Specifically, lymphocyte counts were significantly lower in stage B1 dogs compared with control dogs, whereas total WBC, neutrophil, and monocyte counts were significantly increased in stages C and D. These findings suggest a shift from early immune modulation to overt systemic inflammation as MMVD progresses, consistent with neurohormonal activation and stress-related leukocyte redistribution reported in advanced heart failure [33].

Although the median values in stage C remained within reference limits, leukocytosis and neutrophilia in stage D suggest systemic inflammation in late-stage disease. These findings have shown that CRP is elevated in more advanced stages of heart failure (Figure 3). The interaction between CRP and other inflammatory markers has been documented but is less explored in veterinary cardiology. CRP, an acute-phase protein synthesized in response to cytokines such as IL-6 and TNF-α, often rises in conjunction with neutrophilia and lymphopenia, both of which are observed in our cohort, especially in late-stage disease. Previous reports have shown that elevated CRP levels coincide with altered leukocyte counts, platelet activity, and oxidative stress, all of which promote endothelial injury and remodeling. In our study, leukocytosis and neutrophilia were most apparent in stage D, consistent with systemic inflammation.

### Inflammation and echocardiographic remodeling

The echocardiographic findings were consistent with the expected progression of MMVD, with dogs in later stages showing pronounced enlargement of LA and left ventricle. Interestingly, both NLR and CRP showed significant correlations with LA/Ao and LVIDdN, suggesting that inflammatory activation increases as the heart undergoes remodeling. These findings support the idea that inflammation may not only accompany structural changes but could also contribute to the progression of remodeling in some dogs.

Although the MPA/Ao ratio did not reach statistical significance across MMVD stages, it demonstrated a progressive numerical increase with advancing disease severity. Given that MPA/Ao is commonly used as an echocardiographic surrogate marker for PH, this upward trend likely reflects increasing pulmonary vascular load in more advanced stages. The absence of statistical significance may be attributable to the relatively small number of stage D dogs and variability within groups. Nevertheless, the significant enlargement of absolute MPA diameter supports the presence of progressive pulmonary vascular remodeling. Together, these findings reinforce the contribution of pulmonary vascular changes to disease progression and may help explain the reduced survival observed in dogs with PH. In addition, PH was also associated with higher inflammatory markers. This aligns with literature indicating that pulmonary vascular remodeling and right-sided pressure overload can stimulate inflammation [31]. Although the present study does not establish causation, the observed association suggests that inflammatory markers could help identify dogs at risk for developing PH as part of MMVD progression.

### Diagnostic and prognostic implications

ROC analysis was performed to evaluate the ability of inflammatory markers to discriminate dogs with PH from those without PH. CRP demonstrated moderate diagnostic performance, with an AUC of 0.7517 (95% CI should be inserted if available), which was statistically significant (p = 0.0008). In contrast, NLR showed poor discriminatory capacity, with an AUC of 0.5568 and a non-significant p = 0.3976).

These findings indicate that CRP has measurable diagnostic utility for identifying PH in dogs with MMVD, whereas NLR does not appear to provide clinically meaningful discrimination in this cohort.

Survival analysis showed that dogs with higher NLR and CRP values had poorer outcomes. This supports the use of these markers not only as disease indicators but also as prognostic tools. Because both measurements are inexpensive and widely available, they may serve as practical additions to standard monitoring programs in canine cardiology.

### Study implications and limitations

Overall, the present findings support the concept that systemic inflammation accompanies MMVD progression and is associated with structural cardiac remodeling and clinical outcomes. However, given the retrospective design and the modest strength of observed correlations, further prospective studies are warranted to clarify the mechanistic links between inflammation, atrial remodeling, and PH in dogs with MMVD.

Previous studies investigated the role of CRP in canine MMVD [32]. Subclassifying MMVD stages into C and D enabled a more precise analysis of the diagnostic and prognostic role of CRP and NLR. In this recent study, the pulmonary edema correlation suggests their relevance as prognostic indicators. Our findings provide novel evidence supporting the use of CRP levels and NLR as predictors of PH in dogs with MMVD. Given the need for practical and affordable diagnostic tools in clinical settings, especially where comprehensive testing is limited, CRP and NLR may prove highly beneficial. A key limitation of this study is its retrospective design, which may introduce inherent biases and limited uniform follow-up, and prevent precise control of follow-up intervals.

Limitations of the present study are: (1) this study relied on retrospective records, laboratory data, and echocardiography imaging, which were limited by the consistency of past documentation. (2) Echocardiographic examinations performed by multiple clinicians may have contributed to measurement variability. (3) Biomarker assays were derived from routine diagnostics rather than specialized cytokine panels, which may affect direct comparison with studies using advanced inflammatory profiling. (4) A small number of dogs in stages B1 and D were included, which may have limited statistical power to detect differences among groups. This reflects the clinical population encountered in referral settings, where early and terminal-stage MMVD cases are less represented. (5) The average age of the dog population in this study was old, and some data, e.g., systemic illness, body condition score, and oxidative stress level, may help to reflect the correlation between inflammatory status and cardiac status. In addition, (6) environmental and nutritional conditions could not be controlled, and medical therapy is a potential confounding factor in these populations.

## CONCLUSION

This study demonstrated that systemic inflammatory biomarkers, particularly CRP and NLR, are associated with disease severity and cardiac remodeling in dogs with MMVD. A progressive increase in CRP and NLR was observed with advancing MMVD stages, accompanied by alterations in leukocyte profiles. Echocardiographic findings confirmed that LA enlargement, LA/Ao ratio, LVIDd, and MPA diameter increased with disease progression, indicating progressive cardiac and pulmonary vascular remodeling. Among the biomarkers evaluated, CRP showed moderate diagnostic performance for identifying PH (AUC = 0.7517, p < 0.001), whereas NLR demonstrated limited discriminatory ability. Furthermore, both CRP and NLR were associated with survival outcomes, with higher values corresponding to reduced survival probabilities, particularly in dogs with concurrent PH.

The strength of this study lies in the integration of hematologic, biochemical, and echocardiographic data within a clinically relevant population of dogs with MMVD across multiple ACVIM stages. The use of routinely available biomarkers such as CRP and NLR enhances the translational value of the findings, as these markers can be easily incorporated into standard clinical practice without additional cost or specialized testing. In addition, stratified analyses based on PH status, age, and sex provide a more comprehensive understanding of the interactions between inflammation and cardiac remodeling.

However, the findings should be interpreted in light of certain limitations. The retrospective design may introduce selection bias and variability in data recording, and the absence of a healthy control group restricts comparisons to within-disease categories. Variability in clinical management and echocardiographic assessment may also have influenced the results. Despite these limitations, the observed associations provide meaningful insight into the role of inflammation in MMVD progression.

Future studies should focus on prospective longitudinal designs with standardized follow-up intervals to better define causal relationships between CRP, NLR, and cardiac remodeling. Incorporation of additional inflammatory mediators and advanced imaging techniques may further clarify the underlying mechanisms linking systemic inflammation to structural and functional cardiac changes. Moreover, evaluating the impact of therapeutic interventions on CRP and NLR dynamics could help establish their utility as monitoring tools in clinical cardiology.

In conclusion, CRP and NLR are practical adjunct biomarkers that reflect systemic inflammation and are associated with cardiac remodeling and survival in dogs with MMVD. While echocardiographic parameters remain the primary tools for diagnosis and staging, the inclusion of CRP and NLR may improve risk stratification and clinical decision-making. Their application should be considered complementary rather than substitutive, and interpreted alongside established clinical and imaging findings for optimal patient management.

## DATA AVAILABILITY

The data generated during the study are included in the manuscript.

## AUTHORS’ CONTRIBUTIONS

SP: Conceptualization, methodology, formal analysis, investigation, writing – original draft, writing – review and editing, visualization, and supervision. CP, SS, RT, KS, and SH: Methodology and investigation. All authors have read and agreed to the published version of the manuscript.
